# Supplementary material for: In situ real-time gravimetric and viscoelastic probing of surface films formation on lithium batteries electrodes
Source: Nat Commun. 2017 Nov 9;8:1389. doi: 10.1038/s41467-017-01722-x (PMC5680218; doi:10.1038/s41467-017-01722-x)
Supplement: Supplementary file 1 — Supplementary Information [file 41467_2017_1722_MOESM1_ESM.pdf]

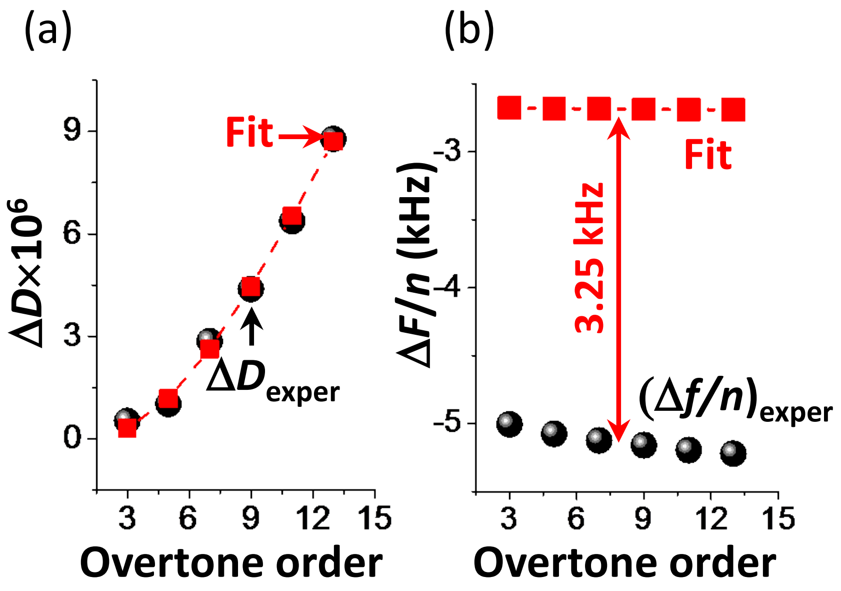

Supplementary Figure 1. Characterization of LTO electrode in air. Overtone-order dependence of dissipation factor and frequency changes of the electrode in air denoted by solid black spheres and fitting to viscoelastic Voigt-type model shown by the red squares. The returned viscoelastic parameters were found to be (subscript "e" denotes electrode): G'_e = 1650 kPa; η_e= 0.0815 kg/ms; h_e= 143 nm; d_e= 3296 kg/m^{3}. Red arrow in panel (b) designates frequency shift 3.25 kHz due to mass effect of the rigid bottom layer.

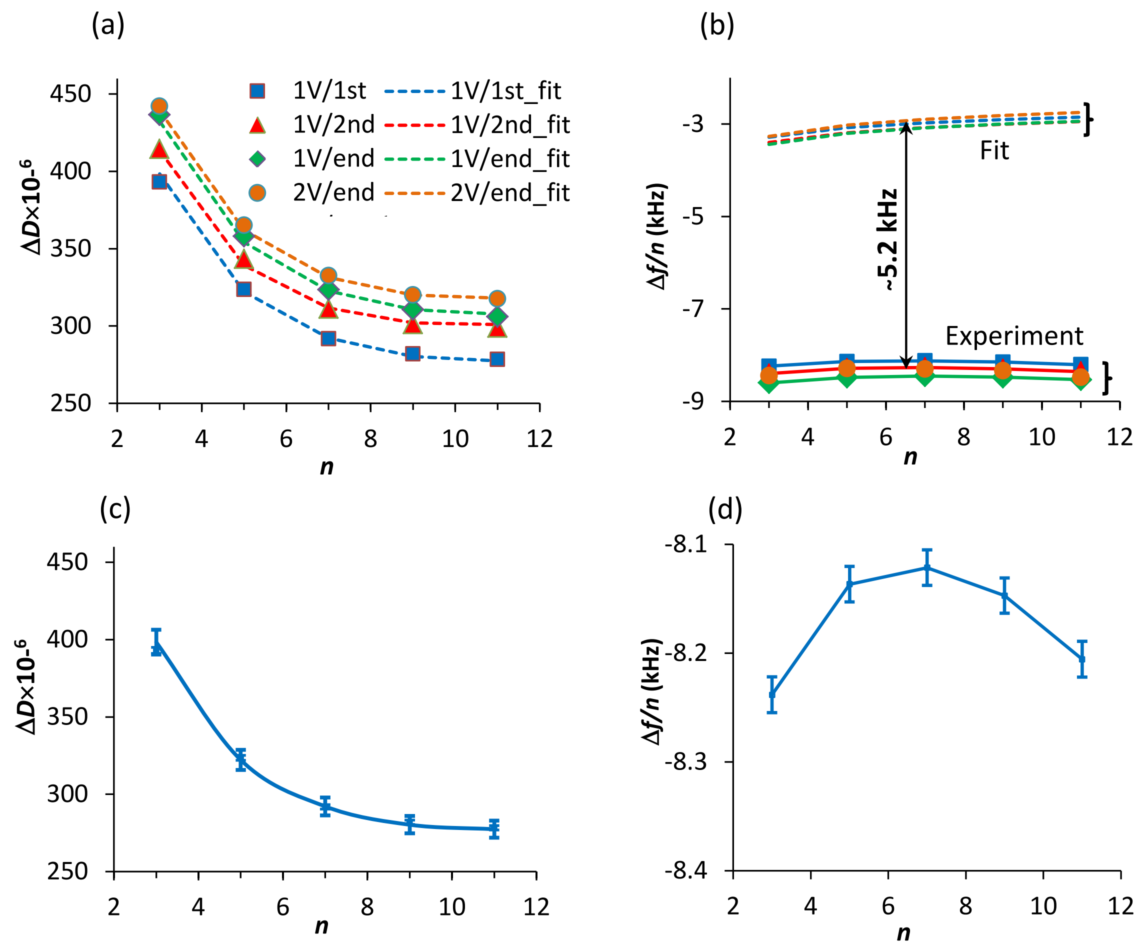

Supplementary Figure 2. Characterization of LTO electrode in 1M LiTFSI solution. Panels (a) and (b) show the Δ*f*/*n* and Δ*D* changes at the selected potentials as indicated by colored symbols. The experimental values were referenced to the state of uncoated crystal . Dashed lines of the same color represent the best fit to the related experimental data.

Panels (c) and (d) show Δ*f*/*n* and Δ*D* changes for the first measurement at 1 V performed with 3 physically different LTO electrodes with their dry mass close to 88 mg/cm^2. The error bars characterize reproducibility of measurements of Δ*f*/*n* and Δ*D* changes at each overtone order. Fitting was performed as described in Methods section. The full set of returned parameters at all selected potentials is listed in Supplementary Table 1.

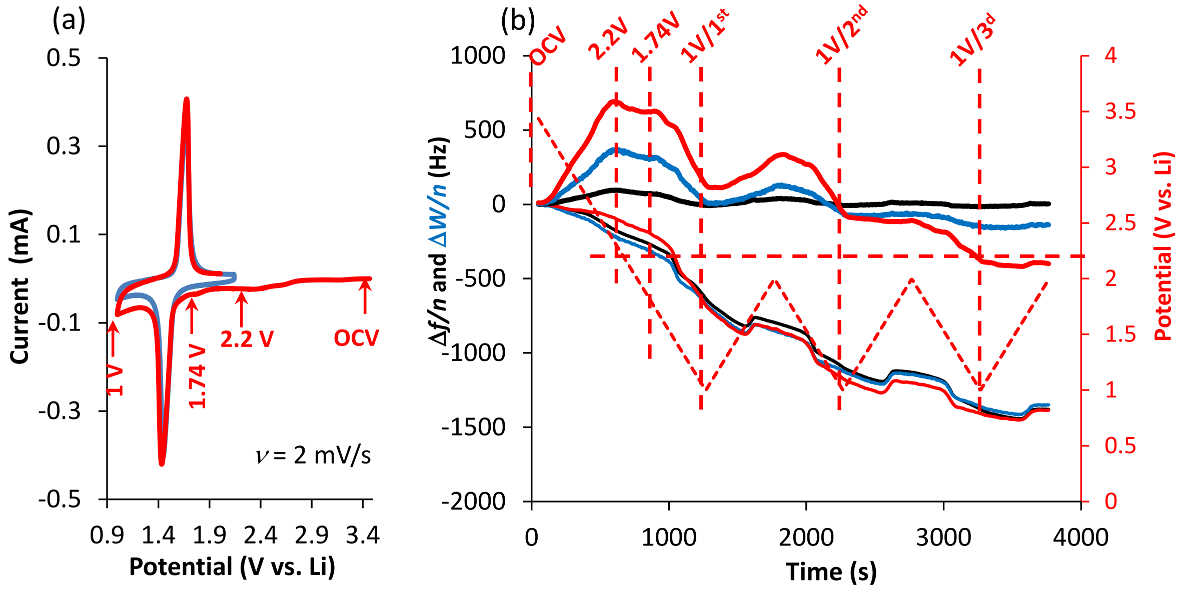

Supplementary Figure 3. Characterization of LTO electrode in 1M $LiPF_6$ + 2% VC solution. (a) Two first sequential CVs from OCV to 1 V and then to 2.2, 1.74 and 1.0V V as indicated.; (b) Frequency and resonance width changes at different overtones (shown by different color) as functions of time during electrode cycling. Dashed red lines denote potentials at which viscoelastic analysis has been carried out.

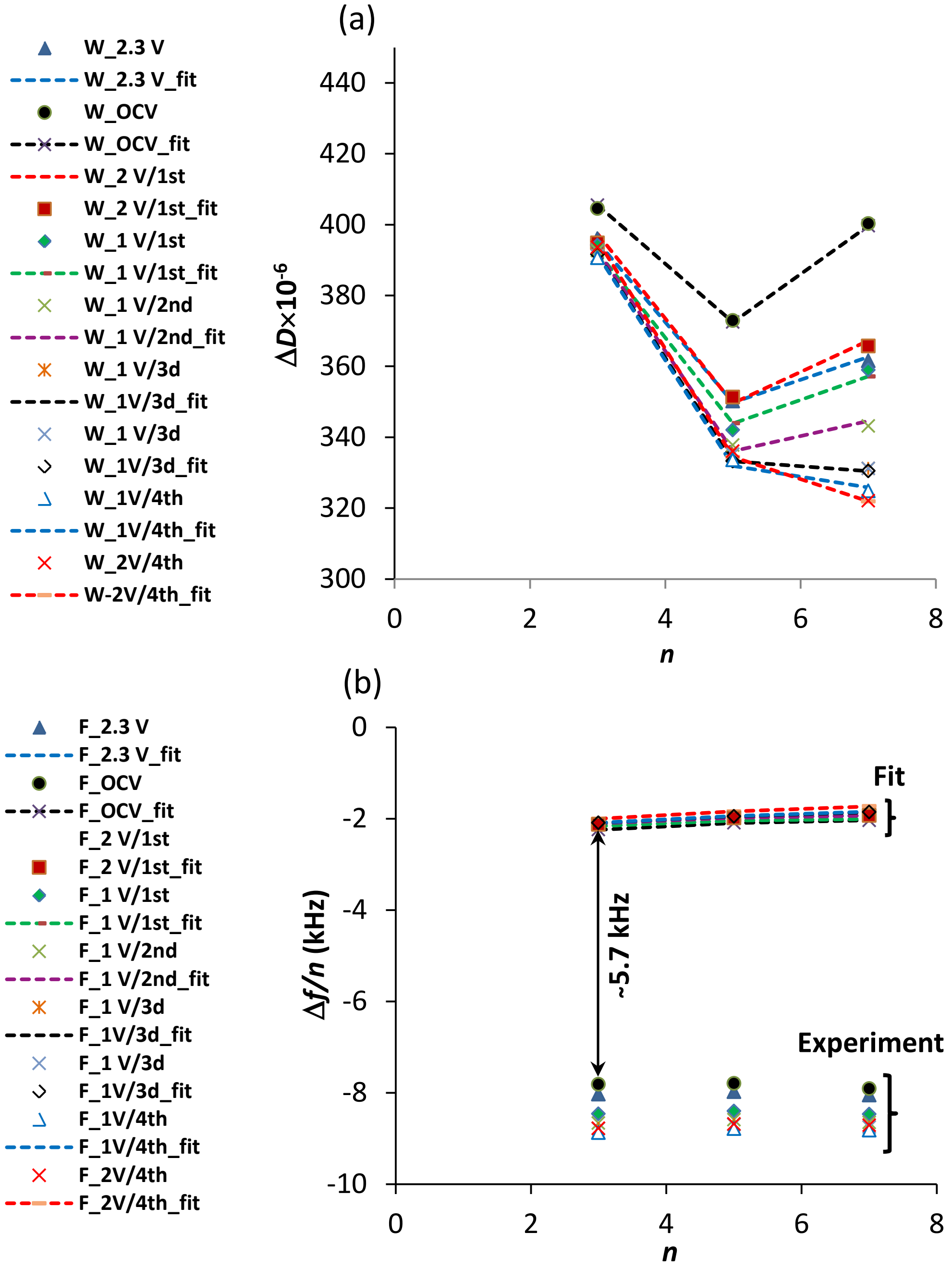

Supplementary Figure 4. Characterization of LTO electrode in 1M $LiPF_6$ solution. Panels (a) and (b) show experimental dissipation and frequency changes at the selected potentials as indicated by colored symbols. The experimental values were referenced to the state of uncoated crystal. Dashed lines of the same color represent best fit to the related experimental data. Fitting was performed as described in Methods section. The full set of returned parameters at all selected potentials is listed in Supplementary Table 2.

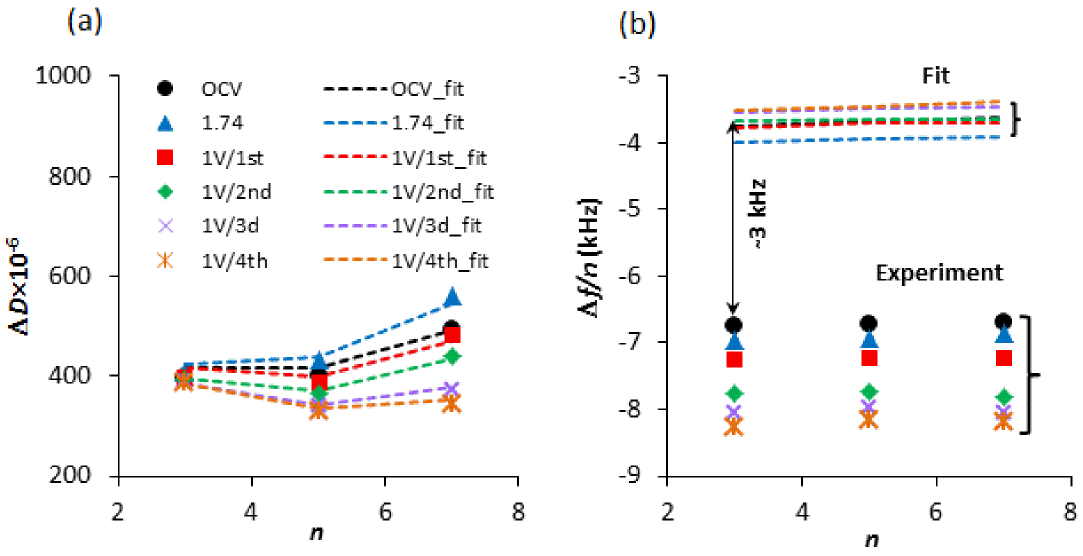

Supplementary Figure 5. Characterization of LTO electrode in 1M $LiPF_6$ + 2% VC solution. Panels (a) and (b) show experimental dissipation and frequency changes at the selected potentials as indicated by colored symbols. The experimental values were referenced to the state of uncoated crystal. Dashed lines of the same color represent best fit to the related experimental data. Fitting was performed as described in Methods section. The full set of returned parameters at all selected potentials is listed in Supplementary Table 3.

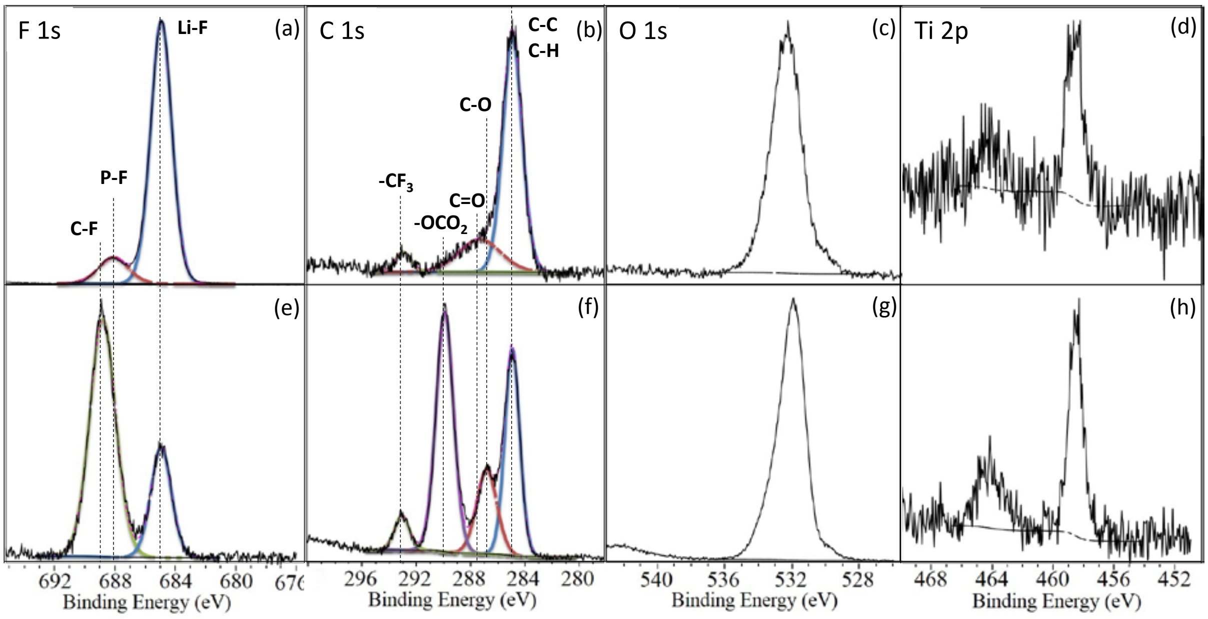

Supplementary Figure 6. XPS of F(1 s), C(1 s), O(1 s), and Ti(2p) measured for LTO electrodes. Solution composition: 1M $LiPF_6$ (a-d), and 1M LiTFSI (e-h).

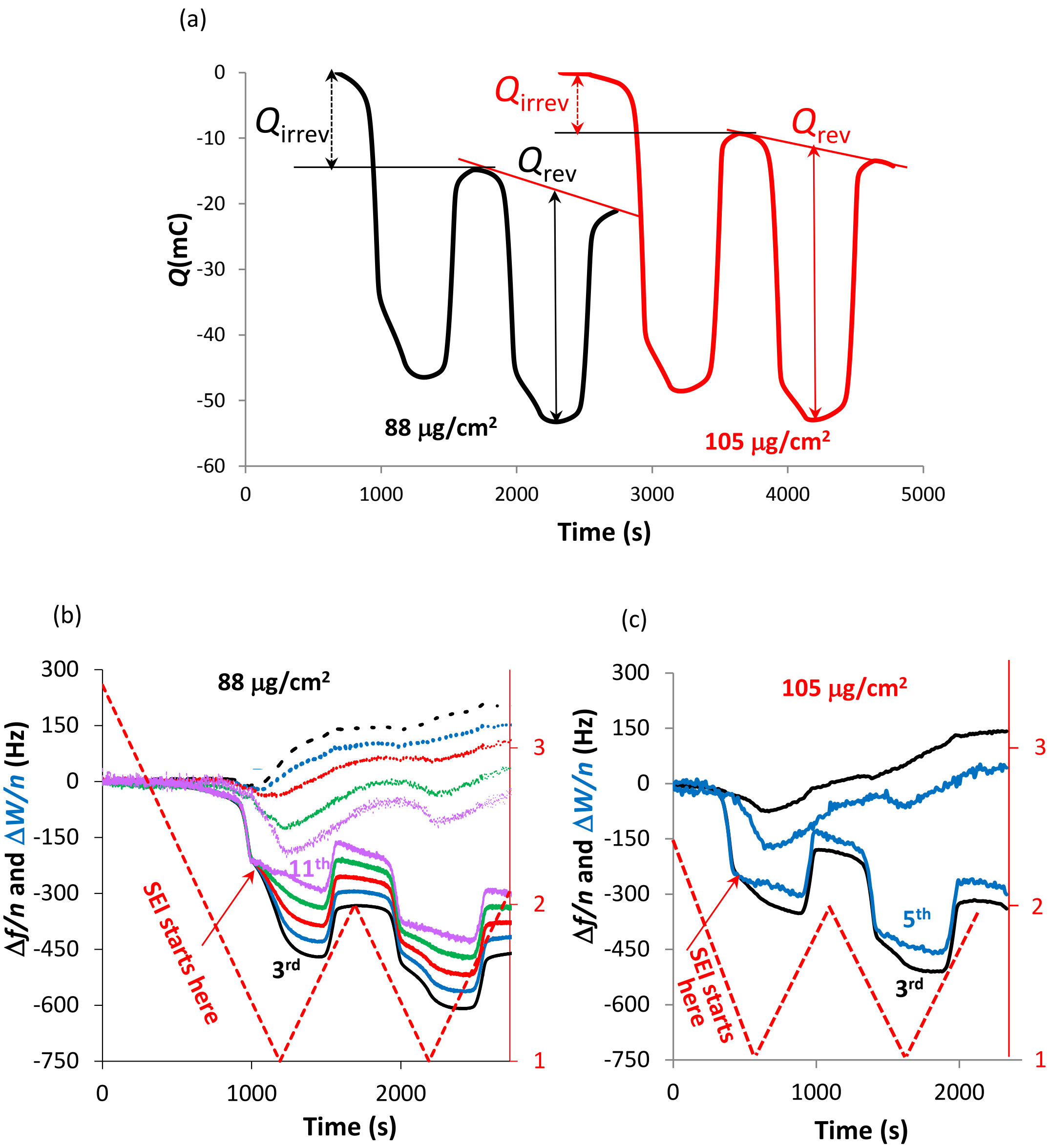

Supplementary Figure 7. Comparison between the charges passed during two first cycles with the use of LTO electrodes with two different loading mass densities (as indicated) measured in 1M LiTFSI solution. The reversible and irreversible charges are shown by the arrows (a). Panels (b) and (c) show the related $\Delta f/n$ and ΔW changes. Note that as the loading mass density becomes larger, only the lower harmonics can be measured.

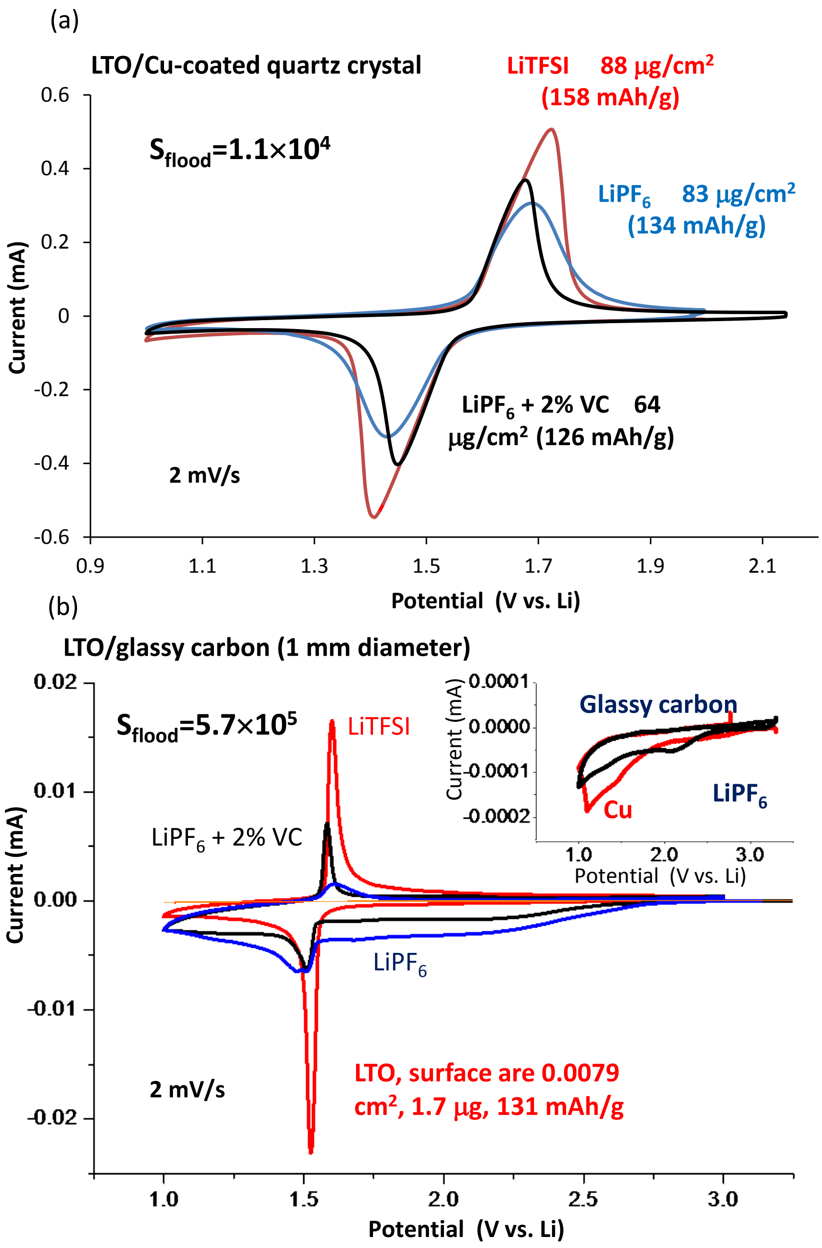

Supplementary Figure 8. Cyclic voltammogramsms measured at 2 mV/s with three LTO electrodes in different electrolyte solutions as indicated. Mass loading density mas measured by EQCM-D in the air, and the intercalation charge (mAh units) divided by the respective masses to calculate practical specific capacity of the electrodes (a).

Similar measurement with the LTO electrodes deposited onto glassy carbon disk electrode 1 mm in diameter. The largest floodness factor (5.7×10^5).deteriorates reversible intercalation capacity in the order LiTFSI<$LiPF_6$+VC<$LiPF_6$ (b).

Supplementary Table 1. Parameters for LTO/PVdF in LiTFSI (density and dynamic viscosity of solution were taken as d_{sol} =1300 kg m^{-3}, η_{sol} =0.00288 kg/ms, respectively).

**Potential**	**Viscoelastic parameters of SEI**				**Viscoelastic parameters of electrodes**			
	G'_s/kPa	η_s /kg/ms	h_s /nm	d_s / kg m^{-3}	G'_e/kPa	η_e, kg/ms	h_e /nm	d_e, kg/m^3
OCV (no SEI)	-	-	-	-	1650	0.0815	143	3296
1.35 V(no SEI)	-	-	-	-	1650	0.0815	143	3296
1V/1st	8.0	0.00016	2.5	2000	1650	0.0815	143	3500
1V/2nd	47	0.0012	23	2000	1660	0.0815	143	3500
1V/3d	68	0.0012	20	2000	1660	0.0815	143	3500
1V/4th	71	0.0012	20	2000	1660	0.0815	143	3500
1V/5th	71	0.0012	20	2000	1660	0.0815	143	3500
1V/6th	72	0.0012	20	2000	1660	0.0815	143	3500
1V/7th	74	0.0012	20	2000	1660	0.0815	143	3500
2V/7th	74	0.0012	20	2000	1660	0.067	143	3296

Supplementary Table 2. Parameters for LTO/PVdF in 1M $LiPF_6$ ((d_{sol} = 1200 / kg m^{-3}, η_{sol} =0.004 kg/ms)

Potential	Viscoelastic parameters of SEI				Viscoelastic parameters of electrodes			
	G'_s,kPa	η_s, kg/ms	h_s, nm	d_s, kg/m^3	G'_e,kPa	η_e, kg/ms	h_e /nm	d_e, kg/m^3
OCV (no SEI)	-	-	-	-	3150	0.003	70.4	
2.3V(SEI)	2.0	0.00155	38	2300	3150	0.003	70.4	3296
2V/1st	4.5	0.00155	32	2300	3150	0.003	70.4	3296
1V/1st	9.5	0.00149	43	2300	3150	0.003	70.4	3500
1V/2nd	18.5	0.00145	61	2300	3150	0.005	70.4	3500
1V/3rd	18.5	0.00147	65	2300	3150	0.009	70.4	3500
1V/4th	18.5	0.00147	65	2300	3150	0.01	70.4	3500
2V/4th	18.5	0.00146	65	2350	2800	0.011	70.4	3296

Supplementary Table 3. Parameters for LTO/PVdF in 1M $LiPF_6$ + 2% VC (d_{sol} =1200 kg m^{-3}, η_{sol} =0.004 kg/ms, for 2.2 V/1st, η_{sol} =0.0042 kg/ms).

Potential	Viscoelastic parameters of SEI				Viscoelastic parameters of electrodes			
	G'_s,kPa	η_s ,kg/ms	h_s, nm	d_s, kg m^{-3}	G'_e,kPa	η_e, kg/ms	h_e /nm	d_e, kg/m^3
OCV (no SEI)	-	-	-	-	9000	0.022	150	3296
2.2V/1st	12	0.065	29	1700	9000	0.022	150	3296
1.74V/1st	12	0.065	20	2000	9000	0.022	150	3296
1V/1st	15	0.0016	35	2000	9000	0.022	150	3500
1V/2nd	19	0.0015	62	1900	9000	0.022	150	3500
1V/3rd	31	0.0013	80	2000	9000	0.036	150	3500
1V/6th	33	0.0013	84	2000	9000	0.048	150	3500

Supplementary Note 1: *Viscoelastic Voigt-type models*

As has been previously mentioned, EQCM-D deals with two output characteristics: the resonant frequency, *f*, normalized by the overtone order (*n*), *f/n*, and the dissipation factor, *D*, defined as the ratio of the full resonance peak width, *W*, to the resonant frequency, *f*: $D = (W/n)/(f/n)$. When the electrode coating is rigidly attached to the crystal surface and the measurements are performed in the gas/air environment, the resonance frequency and the resonance width (or dissipation) is different from those of the neat (uncoated) crystal. The case in which the resonance frequency changes but the dissipation remains constant is considered as a purely gravimetric one. This implies that the entire loading mass (coating's bulk) is entirely stiff and rigidly attached to quartz crystal surface (no-slip condition):in this case the electrode coating moves at the same velocity as the crystal surface itself during crystal's oscillation. The change in the frequency, ($\Delta f/n$), is thus translated into the change in mass, (Δm), using the Sauerbrey's equation: $\Delta m = -C\Delta f/n$, where C is the mass sensitivity constant dependent on the mechanical properties of quartz only. [1] However, the LTO electrodes in air reveal an *n*-dependent change in ΔD and $\Delta f/n$ (Supplementary Figs. 1a and 1b, respectively). This implies that in EQCM-D, the LTO coating should be treated as a viscoelastic rather than the rigid one.

The specificity of the use of viscoelastic models for *particulate composite electrodes* compared to the classical *continuous uniform films* is due to a complicated co-existence of the rigid and viscoelastic layers caused by non-uniform distribution of binder. Note that in the practical composite electrodes, e.g. 50 μm thick and thicker electrodes, the binder is uniformly distributed if the preparation of the electrode slurry was correct. The thickness of the practical electrodes is much larger than the size of the intercalation particles and the pieces of the binder among them. In the electrode-coated quartz crystals the binder is distributed non-uniformly not only because of the features of air brush method distributing diluted electrode slurry, but also

due to the fact that the electrode thickness exceeds the intercalation particles size by only a factor of 2-4 at most. Under these circumstances in view of a smaller mass fraction of the binder with respect to the intercalation particles, the binder is distributed non-uniformly having larger content closer to the quartz crystal surface. Not surprisingly, the type of EQCM-D response depends considerably on the particle size. [2] These large species are bound to the quartz crystal surface not enough strong compared to the small particles which are completely embedded into the 3D polymeric binder network. For this reason large particles experience sliding friction along the oscillating crystal surface enabling a new application of EQCM-D in the energy storage field – as a probe of the binder's adhesion strength to the current collector. [2] As noted previously, viscoelasticity is understood here effectively, as a reduced rigidity and increasing loss of oscillation energy due to the insufficient amount of binder around the top layer, forcing it to move at a different velocity than that of the rigid bottom layer.

From the consideration of LTO electrodes in electrolyte solutions under OCV, we move to the electrodes' polarization, which results in nucleation and growth of SEI-type surface films on the top of the electrode viscoelastic layer. Whether SEI of varying thickness is rigid or viscoelastic is established by direct modeling. The distribution of the velocity profile, in this case, is shown in Fig. 1f. It can be seen that the formation of SEI is considered here as a part of the acoustic multilayer formalism, [3-7] that is, as the formation of a thin viscoelastic layer of SEI in direct contact with the viscoelastic top electrode layer and semi-infinite Newtonian liquid on the opposite side (Fig. 1f).

Fitting this model to the experimental frequency and dissipation changes can be easily done using the commercial software Qtools (as specified below), supplied together with an EQCM-D instrument. This allows evaluation of the solid-layer density, d_s, thickness, h_s, shear storage

modulus, G', and loss modulus, G'' (or, equivalently, solid viscosity, $\eta_S = G''/2\pi n f_o$). Thus, for a single viscoelastic layer in contact with the gas phase, $\Delta f/n$ and ΔD depend on four parameters. Through a built-in menu, the user chooses the fitting (modeling) of a single viscoelastic layer in the gas phase. If another viscoelastic layer is in contact with the first one, another four characteristic parameters are added. Finally, if the top viscoelastic layer is in contact with Newtonian liquid (electrolyte solutions), the menu proposes to add a liquid layer with two additional parameters, namely, liquid's density, d_{liq}, and viscosity, η_{sol}. Formally, viscoelastic modeling resembles modeling of electrochemical impedance spectra.[8] Electrochemical impedance of intercalation-type electrodes implies the probing of various electric interfacial and bulk-electrode properties by a variation of the frequency of *ac* current, whereas in viscoelastic modeling, the mechanical (acoustic) characteristics of solid multi- layers in contact with an electrolyte solution are probed by varying wavelengths of shear waves on the various overtones. Hence, the formation of SEI on the electrode surface results in characteristic changes in the experimental shifts of ΔD and $\Delta f/n$ as a function of the potential. The related parameters are identified by the fitting routine.

Performing viscoelastic modeling, both in gas phase and in liquids (the related velocity profiles are shown in Figs. 1e and 1f), we used Qtools software, whereas the stack of rigid and viscoelastic electrode layers requires fitting first only ΔD vs n dependence. From this dependence, the parameters of the viscoelastic layer are evaluated for their further using to calculate the related values of $\Delta f/n$ for this viscoelastic layer. The values of $\Delta f/n$ for the rigid layer are calculated by subtracting $\Delta f/n$ of the viscoelastic layer from the experimental values of $\Delta f/n$. For this reason, we used general forms of the equations describing the acoustic properties of a single viscoelastic coating in air (Eq. 10.1.4 p. 222) [7] and for two viscoelastic layers, the top of which is in contact with the liquid phase (Eq. 10.6.1 p. 236).[7] With the use of the Maple

software, ΔD vs n were selectively fitted and the viscoelastic parameters were identified. By using these parameters $\Delta f/n$ values were calculated both in Maple and Qtools which appeared to be identical. We thus have confirmed that Qtools uses in its calculation routine Eqs. 10.1.4 and 10.6.1 in their general form (rather than their thin-layer limit).

Supplementary Note 2: *Optimal electrode mass density for sensing SEI formation*

The important feature of EQCM-D characterization of particulate composite electrodes with their effective thickness only slightly larger than the average particle's size, is that there is a limited range of electrodes' mass loadings (and hence thicknesses) suitable for the most sensitive probe of the electrodes' viscoelasticity.

As has been clarified in the main text, too small mass loadings severely deteriorate the reversible capacity of the electrode in view of the increase of the floodness factor, S_{flood}. On the other hand, an increase in the loading mass implies a fortunate decrease in the S_{flood}. However, for not very small intercalation particles the increase in the mass loading (i.e. in the number of intercalation layers), means a gradual increase in the dissipation factor. Higher overtones with lower wavelength of sound feel viscoelasticity (softness) first with the corresponding increase in the dissipation factor above the standard limit: these overtones are not experimentally measured at all. Reduction of the number of relevant overtones below three implies that viscoelastic parameters cannot be retrieved.

A typical example of exceedingly high viscoelasticity, when the electrode mass density increases by 20% from 88 to106 $\mu g/cm^{2,}$, is presented in Supplementary Fig. 8. This figure shows that the reversible capacity of the electrode increases, the extent of the irreversible capacity decreases during the increase of the electrode mass density. However, the thinner electrodes were

characterized by EQCM-D using 5 overtones, (Supplementary Fig. 8b) whereas the thicker electrodes were measured by only two lower overtones (3rd and 5th), see Supplementary Fig. 8c. Note that the beginning of the SEI formation appears at the same place on the $\Delta f/n$ curve, the direction of the changes of $\Delta f/n$ and ΔD with n is also the same, however, the dispersion of the related values with n is different implying the different viscoelasticity of the two types of electrodes (thin or thick active masses).

$LiPF_6$-based solutions which result in the formation of low-quality, thick and porous SEI on LTO surface diminishing the mechanical strength of adhesion of particles to each other exhibits exceedingly high viscoelasticity. It is for this reason we selected the loading mass of the LTO electrode for testing in $LiPF_6$ solution, 83 $\mu g/cm^2$, only slightly smaller than that for the LiTFSI solution, 88 $\mu g/cm^2$. However, in $LiPF_6$ + 2% VC solution the larger initial extent of the reaction with VC results in the effective softness of LTO electrode suppressing oscillations on higher harmonics. It is for this reason the optimal mass of the electrode in this solution was reduced down to 64 $\mu g/cm^2$.

Supplementary Note 3: *Statistical errors of EQCM-D measurements*

The frequency shift in the air due to the "dry" electrode mass density for the measurements in LiTFSI, $LiPF_6$ and $LiPF_6$+2% VC were 5.0, 4.7 and 3.6 kHz corresponding to the following loading mass densities: 88, 83 and 64 $\mu g/cm^2$. A standard precision of QCM-D instrument is $\pm$0.1 Hz. However, the precision of fabrication of dry electrode coatings is limited by the use of the manual airbrush spraying technique. From 3-6 attempts the best precision in frequency shift that we could typically reach was $\pm$ 15 Hz, for example, the frequency change for a dry LTO coating further tested in LiTFSI solution was 5000Hz $\pm$ 15 Hz.

The dry mass of the coating is obtained from the difference of the frequency of the neat (uncoated) crystal and the frequency of the coated crystal. Here another limitation of the precision related to the different static stresses on the crystal during its mounting to the cell via o-ring is approached. The measurement of the frequency change of the dry coating implies disassembling of the cell after measurement of the neat crystal, and then its further assembling for the measurement of the coated crystal. Reassembling of the cell typically results in a random frequency shift of the same order of magnitude as the electrode fabrication, ±15 Hz. If reassembling of the cell is not involved, e.g. deposition of thin metallic films in a vacuum takes place, the precision increases by 2 orders of magnitude.

Let us consider the precision of frequency measurements of a dry coating after its immersion into liquid (electrolyte solution). For example, for the 5 kHz (frequency shift in air) LTO electrode coating obtained on 3rd overtone, after its immersion into a LiTFSI solution, the frequency shift with respect to that of the neat crystal in the air for 3-4 samples is equal to 8240 Hz± 20 Hz. This good precision was reached only after the introduction of impregnation of the coated crystal in solution under vacuum (as is usually done for thick electrodes for measurements in coin cells). Assuming that reassembling of the cell ensures the precision of ± 15 Hz, the minor remaining contribution, ± 5 Hz, should be assigned to the variation of the porous structure of the different samples, possible swelling, and other minor effects. Error bars are indicated in Supplementary Fig. 2c, d.

Supplementary References

1 Sauerbrey, G. Verwendung von Schwingquarzen zur Wägung dünner Schichten und zur Mikrowägung. *Z. Phys.* **155**, 206-222 (1959).

2 Dargel, V. *et al.* In Situ Multilength-Scale Tracking of Dimensional and Viscoelastic Changes in Composite Battery Electrodes. *ACS Appl. Mater. Interfaces* **9**, 27664–27675 (2017).
3 Bund, A. & Schneider , M. Characterization of the Viscoelasticity and the Surface Roughness of Electrochemically Prepared Conducting Polymer Films by Impedance Measurements at Quartz Crystals. *J. Electrochem. Soc.* **149**, E331-E339 (2002).
4 Efimov, I. & Hillman, A. R. Correlation of viscoelastic properties with solvation of regioregular poly (3-decylthiophene) films. *Anal.Chem.* **78**, 3616-3623 (2006).
5 Efimov, I., Ispas, A. & Bund, A. Taking into account of surface roughness for the calculation of elastic moduli of polymer films from acoustic impedance data. *Electrochim. Acta* **122**, 16-20 (2014).
6 Eisele, N. B., Andersson, F. I., Frey, S. & Richter, R. P. Viscoelasticity of thin biomolecular films: a case study on nucleoporin phenylalanine-glycine repeats grafted to a histidine-tag capturing QCM-D sensor. *Biomacromolecules* **13**, 2322-2332 (2012).
7 Johannsmann, D. *The Quartz Crystal Microbalance in Soft Matter Research*. (Springer, 2014).
8 Levi, M. D. *et al.* In Situ Porous Structure Characterization of Electrodes for Energy Storage and Conversion by EQCM-D: a Review. *Electrochim. Acta* **232**, 271-284 (2017).
